# Surface Integrity of Dimethacrylate Composite Resins with Low Shrinkage Comonomers

**DOI:** 10.3390/ma14071614

**Published:** 2021-03-26

**Authors:** Jingwei He, Sufyan Garoushi, Eija Säilynoja, Pekka Vallittu, Lippo Lassila

**Affiliations:** 1 Department of Biomaterials Science and Turku Clinical Biomaterials Center—TCBC, Institute of Dentistry, University of Turku, 20520 Turku, Finland; sufgar@utu.fi (S.G.); pekval@utu.fi (P.V.); liplas@utu.fi (L.L.); 2 College of Materials Science and Engineering, South China University of Technology, Guangzhou 510641, China; 3 Research Development and Production Department, Stick Tech Ltd.—Member of GC Group, 20521 Turku, Finland; eija.sailynoja@utu.fi; 4 City of Turku Welfare Division, Oral Health Care, 20101 Turku, Finland

**Keywords:** low-shrinkage monomers, surface gloss, wear, color change

## Abstract

The goal of current research was to investigate the influence of adding low shrinkage “Phene” like comonomers hexaethylene glycol bis(carbamate-isoproply-α-methylstyrene) (HE-Phene) and triethylene glycol bis(carbamate-isoproply-α-methylstyrene) (TE-Phene) on the surface and color characteristics of composite resin. A range of weight fractions (0, 10, 20, 30, 40 wt.%) of HE/TE-Phene monomers were mixed with bisphenol A glycidyl methacrylate (GMA)/triethylene glycol dimethacrylate (TEGDMA) monomer. Experimental composite resins were made by mixing 71 wt.% of silica fillers to 29 wt.% of the resin matrix. A Vickers indenter and glossmeter were used for testing surface hardness (SH) and gloss (SG) at 60°. A chewing-simulator was used to evaluate the surface wear after 15,000 cycles. Color change (∆E) and translucency parameter (TP) were measured using a spectrophotometer. Data showed that HE/TE-Phene monomer had no negative impact (*p* > 0.05) on surface gloss, wear, color change and translucency of experimental composite resins. Surface hardness was in a reducing direction with the increas in HE/TE-Phene weight fraction (*p* < 0.05). The study results suggested that incorporating HE/TE-Phene monomers up to 30 wt.% with Bis-GMA/TEGDMA resin did not negatively influence the surface integrity of composite resins except for SH.

## 1. Introduction

Restorative dental composite resins produced out of a blend of silanized inorganic fillers and methacrylate monomers. Dimethacrylate monomers like triethylene glycol dimethacrylate (TEGDMA), Urethane dimethacrylate (UDMA) and bisphenol A glycidyl methacrylate (Bis-GMA) are the most common used monomers in dentistry [1,2,3]. Formulated composite resin through these monomers has features that fulfil most of practical needs, for instance adequate strength and monomers curing, low water solubility and uptake, superior esthetic and handling characteristics [1]. Despite that, until now, available composite resins in the market still had limitations required to be considered. Certainly, limitations concerned curing contraction stress remain a practical challenge, leading to composite resin fillings failure [4,5,6]. In order to create composite resins with substantial enhancements, formulation of a novel resin approach, unlike the conventional dental resins, had been discussed. From our earlier studies, we showed that the double bonds reactivity in the α-methylstyryl group of a novel Phene monomer was less than the double bond reactivity in a conventional methacrylate group [7,8]. Therefore, the curing rate proceeds slowly in monomer with α-methylstyryl groups than the monomer with the methacrylate group, which offers enough time to alleviate the shrinkage stress by hindering the gel stage during light irradiation [9]. Consequently, utilization of Phene monomer with a small concentration of the double bond was an efficient method to manufacture composite resins with enhanced volumetric shrinkage and shrinkage stress [8]. However, color change of cured composite resins including Phene monomer following storage was noticed, owing to the oxidization of the tertiary amine in the Phene structure [10].

Beside the low shrinkage stress and superior strength, the clinically favorable outcome of composite resin restorations relies primarily on their surface integrity and esthetic properties like hardness, gloss, translucency, color change and wear resistance [1,11]. The long-term clinical success of composite resin restorations is impacted by surface integrity, which can be affected by material formulation and/or deterioration due to exposure in the oral environment [12]. Furthermore, surface integrity has an effect on the material’s resistance to load application and scratch generation [13]. The glossy surface prevents plaque retention and the formation of discoloring films, in addition to enhancing the overall appearance [14]. Furthermore, by enhancing surface hardness and smoothness, the coefficient of friction can be decreased, potentially increasing wear resistance and jeopardizing the composite resin fillings’ survival [1,11]. The quality of brittle composite resins’ surface can also have effects on the load-bearing capacity and fracture behavior [15]. Factors like monomer refraction index, monomer type and crosslinking density of a polymer could influence the surface and esthetic properties of composite resins [11,16,17]. In light of this information and with the target of seeking a novel comonomer that can significantly decrease the shrinkage stress of dimethacrylate monomer without disturbing the surface and color properties of the composite resins, our group evaluated the benefits of using a new comonomers “HE/TE-Phene” (hexaethylene glycol bis(carbamate-isoproply-α-methylstyrene) (HE-Phene) and triethylene glycol bis(carbamate-isoproply-α-methylstyrene) (TE-Phene)) without tertiary amine and with α-methylstyryl structures for developing experimental composite resins [18]. Thus, the aim of this study was to investigate the surface- and color-related properties (surface hardness, surface gloss, wear, color change and translucency) of new experimental composite resins. The research hypothesis was that experimental composite resins would have equal surface-related characteristics when compared with the ordinary dimethacrylate-based composite resin.

## 2. Materials and Methods

### 2.1. Materials

Through Esstech Inc. (Essington, PA, USA) TEGDMA and Bis-GMA were acquired. From Sigma-Aldrich (St. Louis, MO, USA) N,N′-dimethylaminoethyl methacrylate (DMAEMA), and Camphoroquinone (CQ) were acquired. The used reagents were not purified. From Schott (UltraFine, Schott, Landshut, Germany) Silica fillers BaAlSiO_2_ (Ø 0.7 µm) were obtained silanated. The synthesis of “Phene like monomers” (hexaethylene glycol bis(carbamate-isoproply-α-methylstyrene) (HE-Phene) and triethylene glycol bis(carbamate-isoproply-α-methylstyrene) (TE-Phene)) was performed according to previous research [18].

### 2.2. Preparation of the Experimental Composites Resins

Composite resins were made following to the compositions given in Table 1 [18]. The used monomers were weighed and mixed with magnetic stirring. Experimental composite resins were made by mixing silica fillers with each resin matrix using a high-speed mixing machine (SpeedMixer, DAC150 FVZ-K, Hauschild, Germany) with a speed of 1900 rpm. The weight fraction between the fillers and resin’s matrix was 5/2 (wt/wt).

### 2.3. Surface Hardness

Disc shaped specimens (*n* = 5) for each experimental composite resin (Ø 6.5 mm × 2 mm) were prepared. The composite resin was cured using a hand light-curing unit (wavelength = 430~480 nm, light intensity = 1600 mW/cm^2^, Elipar TM S10, 3M ESPE, Seefeld, Germany), and three overlapping portions from one side of the mold were irradiated for 20 s separately. Afterwards, every specimen was polished (grit up to 4000 FEPA (Federation of European Producers Abrasives)) by an automatic grinding machine (Steruers Rotopol-11, Copenhagen, Denmark) at 300 rpm under water cooling. Before testing, all the specimens were stored in dry atmosphere for 24 h at 37 °C. The surface hardness (SH) of every composite resin was measured using a Struers Duramin hardness microscope (Struers, Copenhagen, Denmark) with a 40 objective lens and a load of 1.96 N applied for 10 s. A total of 5 indentations were carried out for each specimen. The diagonal length impressions were measured, and Vickers values were converted into hardness values by the machine. Surface hardness was measured according to the following equation:

(1) $$H=\;\frac{1854.4\times P}{d^{2}}$$

where H is Vickers hardness in kg/mm^2^, d is the length of the diagonals in μm and P is the load in grams.

### 2.4. Surface Gloss

Three block-shaped specimens for each experimental composite resin with a size of 40 mm × 10 mm × 2 mm were prepared in half-split molds between two transparent Mylar sheets. A thin glass plate was placed on the composite resin free surface to remove the material excess. Curing of the composite resin was performed by a hand light-curing unit (Elipar TM S10, 3M ESPE, Seefeld, Germany) for 20 s in ten separate overlapping portions from one side of the mold.

The surface gloss (SG) was measured by a calibrated infrared Zehntner-Glossmeter (GmbH Testing Instruments, Berlin, Germany) at 60° incidence angle with a square measurement area of 6 mm × 40 mm area. The surface gloss was assessed for polished (grit up to 4000 FEPA) and unpolished sides. Every surface was measured five times to obtain the average value.

### 2.5. Two-Body Wear

For localized wear testing, six specimens of each composite resin (n = 6) were prepared in acrylic resin block. Longitudinal cavities with a size of 20 mm × 10 mm × 3 mm were prepared in the composite blocks and then the composite resins were placed in one increment into the prepared cavities and covered with a Mylar strip followed by a glass slide. Subsequently, the composite resins were light irradiated (Elipar TM S10, 3M ESPE, Seefeld, Germany) in five overlapping portions for 20 s separately. Every specimen was then polished using a sequence of #1200 to #4000 grit silicon carbide papers until the surface was flat, and then stored in water for one day at 37 °C. After that, a chewing simulator CS-4.2 (SD Mechatronik, Feldkirchen-Westerham, Germany), which has two chambers simulating the vertical and horizontal movements simultaneously with water, was used to perform the 2-body wear test of specimen. In each of the chamber, there exists an upper sample holder that can fasten the loading tip (antagonistic) with a screw and a lower plastic sample holder in which the composite resin specimen was embedded. Steatite balls with diameter of 6 mm were used as loading tips according to manufacturer’s standard. The balls were embedded in acrylic resins in the upper sample holders and fixed with a fastening screw. A weight of 2 kg that is comparable to 20 N of chewing force was used, and the test was undertaken with 15,000 loading cycles with a frequency of 1.5 Hz.

A 3D optical microscope (Bruker Nano GmbH, Berlin, Germany) was used to profile the wear scratch (n = 6) on each of specimen’s surfaces with Vision64 software (version 1, Bruker Nano GmbH, Berlin, Germany). The maximum wear depth values (µm), representing the average of the lowest or deepest points of all profile scans were calculated from different points.

### 2.6. Color Change (∆E) and Translucency (TP)

Disc shaped specimens (n = 3) for each experimental composite resin (Ø 10 mm × 2 mm) were prepared. Curing of the composite resin was the performed same as before. After curing, the specimens were stored in dry atmosphere (24 h) at room temperature prior to testing.

Specimens’ color was assessed (at day 1,2,3,4,5,6,7) based on CIELAB color scale relative to the standard illuminant D65 over a black tile (CIE L* = 0, a* = 0.01 and b* = 0.03) and a white tile (CIE L* = 99.25, a* = −0.09 and b* = 0.05) on a reflection spectrophotometer (CM-700d, Konica-Minolta, Tokyo, Japan). The size of aperture was Ø 3 mm, and the illuminating and viewing configuration was CIE diffuse/10° geometry with the specular component included (SCI) geometry.

The translucency of the composite resins was obtained by calculating the color difference between the specimen over the white background and the specimen over the black background:

TP = [(L_W_* − _LB_*)^2^ + (a_W_* −a_B_*)^2^ + (b_W_* − b_B_*)^2^]^1/2^ (2)

where the subscript “W” refers to the color coordinates over the white background and the subscript “B” refers to those over the black background.

### 2.7. Statistical Analysis

The results for each tested parameter (SH, SG, wear, ∆E, and TP) were examined by analysis of variance (ANOVA) with SPSS version 23 (SPSS, IBM Corp., New York, NY USA) at the *p* < 0.05 significance level accompanied by a Tukey HSD post hoc test to determine the differences between the groups.

## 3. Results

### 3.1. Surface Hardnes

**Figure 1:**
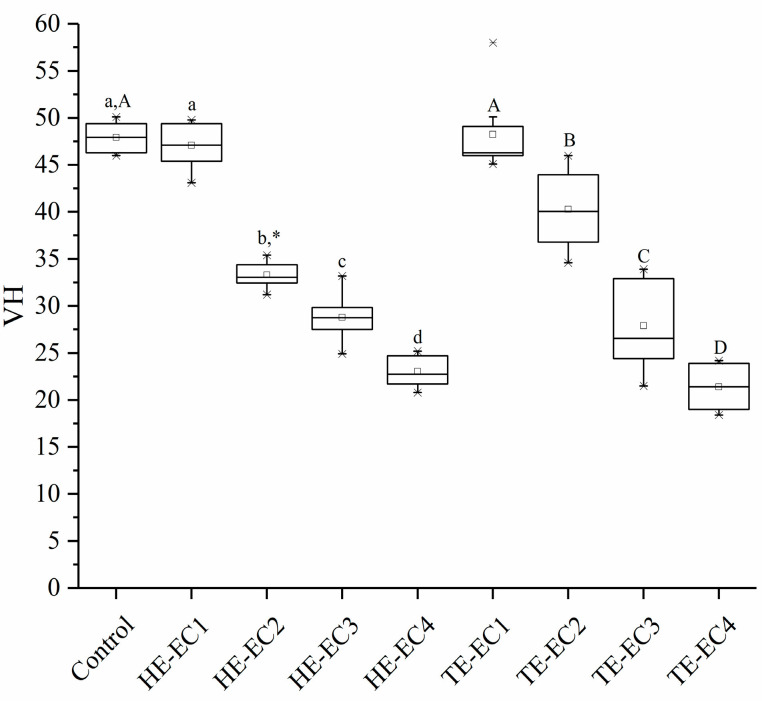
Surface hardness (VH) mean values of experimental composite resins. The same lower letters indicate that there is no significant difference in surface hardness between experimental composite resins with different weight fractions of HE-Phene in the resin matrix. The same upper letters indicate that there is no significant difference in surface hardness between experimental composite resins with different weight fractions of TE-Phene in the resin matrix. The asterisk * indicates the statistical differences between composites with different Phene like monomers at the same mass fraction in the resin matrix.

Figure 1 depicts the results of SH. As shown in Figure 1, SH was in a reducing direction of with the increase in HE/TE-Phene weight fraction (*p* < 0.05) except for HE/TE-EC1, which showed no statistically significant difference from the control (*p* > 0.05). With the same weight fraction, HE-EC had comparable SH to TE-EC (*p* > 0.05), except for HE-EC2, which had lower SH than TE-EC2 (*p* < 0.05).

### 3.2. Surface Gloss

**Figure 2:**
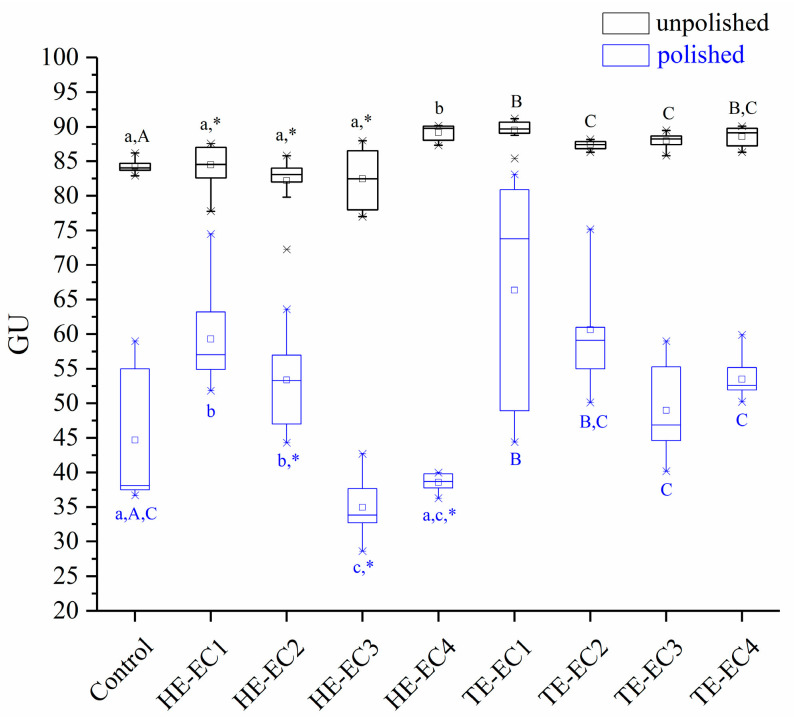
Surface Gloss (GU) mean values of experimental composite resins. The same lower letters indicate that there is no significant difference in surface gloss between experimental composite resins with different weight fractions of HE-Phene in the resin matrix. The same upper letters indicate that there is no significant difference in surface gloss between experimental composite resins with different weight fraction of TE-Phene in resin matrix. The asterisk * indicates the statistical differences between composites with different Phene like monomers at the same mass fraction in resin matrix.

The results of SG are shown in Figure 2, and the results presented that incorporation of HE/TE-Phene had no negative influence on SG of experimental composite resins. Before being polished, HE-EC4, and all TE-ECs had higher SG than the control (*p* < 0.05). All the other HE-ECs had comparable SG to the control (*p* > 0.05). With the same weight fraction, HE-EC had lower SG than TE-EC (*p* < 0.05), except for HE-EC4, which had comparable SG to TE-EC4 (*p* > 0.05). The used polishing protocol reduced the SG of composite resins surfaces when compared to the unpolished surfaces (*p* < 0.05). After being polished, only HE-EC3 had lower SG than the control (*p* < 0.05), HE-EC1, HE-EC2, and TE-EC1 showed higher SG than the control (*p* < 0.05), and all the other HE/TE-ECs had comparable SG to the control (*p* > 0.05).

### 3.3. Wear Depth

**Figure 3:**
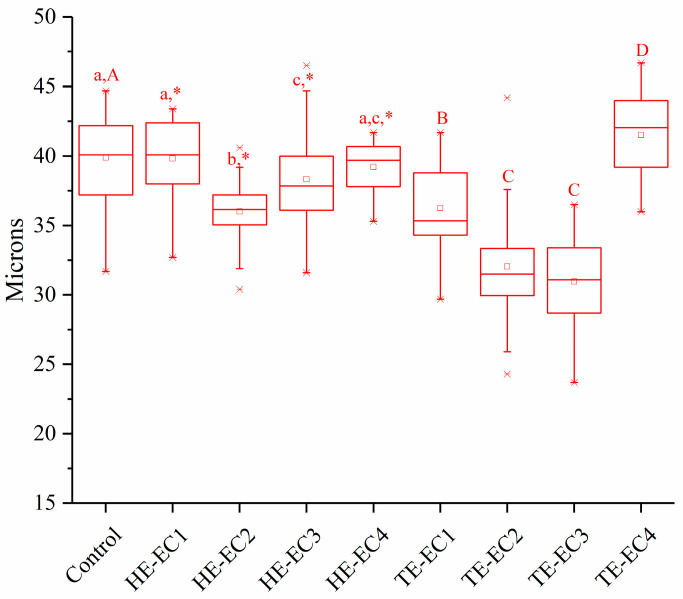
Wear depth mean values (micron) of experimental composite resins after 15,000 cycles of the 2-body wear test. The same lower letters indicate that there is no significant difference in wear depth between experimental composite resins with different weight fractions of HE-Phene in the resin matrix. The same upper letters indicate that there is no significant difference in wear depth between experimental composite resins with different weight fractions of TE-Phene in the resin matrix. The asterisk * indicates the statistical differences between composites with different Phene like monomers at the same mass fraction in resin matrix.

Figure 3 presents the results of the two-body wear test. Compared with the control, only TE-EC4 had higher wear depth (*p* < 0.05), HE-EC1 and HE-EC4 had comparable wear depth (*p* > 0.05), and all the other HE/TE-ECs had lower wear depth (*p* < 0.05). Experimental TE-EC2 and TE-EC3 composite resin presented the lowest values of wear depth (*p* < 0.05) compared with all the tested composite resins. Conversely, TE-EC4 composite resin had the highest values of wear depth compared to all the tested composite resins (*p* < 0.05).

### 3.4. Color Change (∆E) and Translucency (TP)

**Figure 4:**
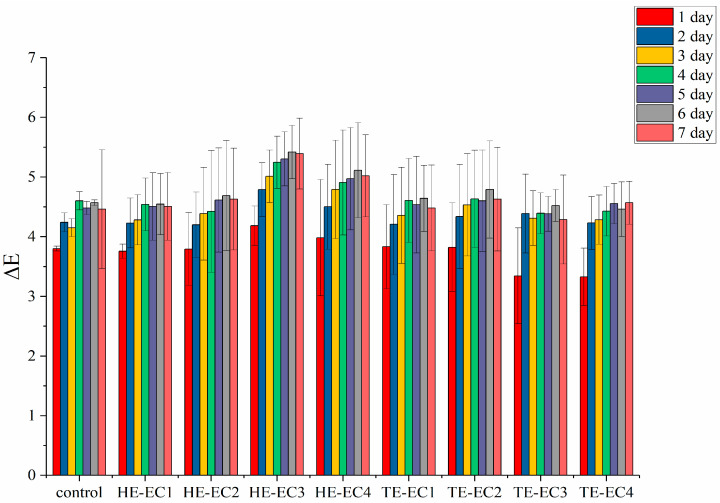
Color change (∆E) mean values of experimental composite resins over one week. Vertical lines represent standard deviation.

**Figure 5:**
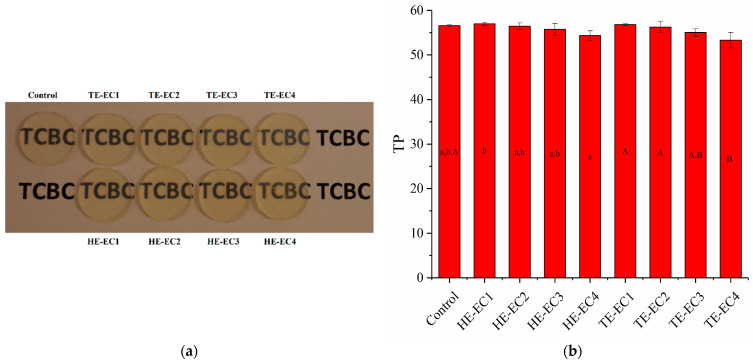
(**a**) Visual image of 2-mm thick composite resin specimens placed over a black letters line. (**b**) Translucency parameter mean values (TP) of experimental composite resins. The same lower letters indicated that there is no significant difference in TP between experimental composite resins with different weight fractions of HE-Phene in the resin matrix. The same upper letters indicated that there is no significant difference in TP between experimental composite resins with different weight fractions of TE-Phene in the resin matrix.

The results of color change (∆E) and translucency (TP) are shown in Figure 4 and Figure 5, respectively. All of the experimental composite resins had comparable values of color change (∆E) during the 7 days except for HE-EC3 and HE-EC4 (Figure 4). Comparing the TP values between all experimental composite resins, only TE-EC4 composite resins showed statistically lower TP values than the control (*p* < 0.05). However, these differences can hardly be recognized visually (Figure 5a).

## 4. Discussion

In our previous research, we presented a new monomer (Phene) with a low double bond concentration and two α-methylstyryl structure [8]. Including Phene monomer in traditional dimethacrylate-based resin gave materials with enhanced volumetric shrinkage and shrinkage stress without disturbing the physical and mechanical characteristics of the composite resins. Despite that, color change of cured composite resins including Phene monomer following storage was noticed owing to the oxidization of the tertiary amine in the Phene structure [10]. In this study, with the intention to enhance the color stability of Phene containing composite resin, high molecular weight Phene-like monomers (HE/TE-Phene) were developed without tertiary amine and with a two α-methylstyryl structure [18]. Data showed that the inclusion of HE/TE-Phene monomers had no negative effect on the most of the tested surface and color characteristics of composite resins. 

Surface hardness was in a reducing direction with the increase in HE/TE-Phene weight fraction (Figure 1). This reduction should be mainly attributed to a decrease in the matrix crosslinking density, which was considered to be the key element for the physical characteristics of composite resins [19,20]. Accordingly, once the weight fraction of HE/TE-Phene was not more than 10 wt.% in a resin matrix, the strengthening impact of benzene groups was compensated by a lowering in matrix crosslinking density, resulting in equivalent SH as conventional composite resin. In order to give successful composite restorations, wear resistance is considered as a principal factor in the selection of composite resin. Curiously, TE-EC2 and TE-EC3 composite resin presented the lowest values of wear depth out of all the tested composite resins (Figure 3), which might be partly explained by the resin matrix resiliency that possibly provides certain shock-absorbing capability. On the other hands, the highest wear depth values were found for TE-EC4, which showed good connection with surface hardness results. However, the rest of the results did not show a correlation between surface hardness and two-body wear, which is in line with some previous findings [21,22].

As stated by the ISO (International Organization for Standardization), semigloss surfaces like composite resins need to be evaluated with an illumination angle of 60 degrees, which was used in our study. The 60° angle was identified to be similar to the angulation where the ordinary person will look at the surface [17]. TE-Phene composite resins presented superior values of SG than HE-Phene composite resins and this might be explained by the lower shrinkage stress of TE-Phene composite resins, which was beneficial for obtaining a smoother surface. In general, higher gloss is associated with smoother surface of dental composites [23]. Following the recommendation of Roeder and colleagues to standardize the specimens, in this research, we evaluated the SG of composite resins after and prior to polishing [24]. The SG values of polished composite resin was significantly lower than prior polishing (Figure 2). A comparable finding was found in the research of Lassila et al. and Cazzaniga and colleagues [14,25]. Despite the fact that light cured composite surfaces touching the Mylar sheet were glossy and smoother, restoration grinding and polishing are often needed in order to reshape the morphology and to eliminate extra composite; this decreases the SG and obliges polishing of composite restoration [26]. Furthermore, the cured composite in touch with the Mylar sheet is full of low polymerized oxygen inhibition resin layer and air bubbles which leads to it wearing easily [27]. 

Translucency (TP) of the composite determines the viewed variation in color among constant width of the composite across a black base and equal width of the composite across a white base. The TP measurements or values are usually equivalent to the optical understanding of translucency; as long as the composite is fully dark or opaque, the TP value is zero [28]. In our study, no difference was observed in TP values among the tested composite resins. Since filler composition and content are same among the tested composite resins, the monomers refraction index matches the filler refraction index. Color stability can be assessed by instrumental and visual methods. The used Spectrophotometry in this study has proven to be a sound method in composite resins research, being capable of suppressing the individual analysis of visible color comparison [29]. All tested composite resins showed similar measurements of color change (∆E) during the 7 days except for HE-EC3 and HE-EC4 (Figure 4). There are several factors may influence the stability of composite resins’ color, such as the component of monomers, type and amounts of initiators and amine accelerators [10,23]. In this study, the different ∆E values of HE-EC3 and HE-EC4 should be due to their different components when compared with other composites.

It is important to highlight that tested surface characteristics of our experimental composite resins were of a similar level to many commercial dental composite resins tested under same laboratory conditions [14,22,30,31]. 

In our study, the limitation was that we studied the surface and optical characterizations in a dry condition and over short time period. In a patient’s mouth, the impact of saliva and temperature changes because of food and drinks should be considered. Furthermore, the composite surface roughness might be altered due to chewing action resulting in deposition and retention of discoloring factors. In further study, long-term water storage, a brushing/abrasive test, and biocompatibility should also be assessed in order to demonstrate the whole picture of this experimental composite resin.

## 5. Conclusions

According to the used experimental conditions, the study results suggested that incorporating HE/TE-Phene monomers up to 30 wt.% with Bis-GMA/TEGDMA resin did not negatively affect the surface integrity of composite resins except for SH.

## Figures and Tables

**Table 1:** Composition of resin matrix for each hexaethylene (HE) or triethylene (TE) experimental resin composite [18].

Resin Matrix	Components (%)				
	Bis-GMA	TEGDMA	HE/TE-Phene	DMAEMA	CQ
**Control**	49.3	49.3	0	0.7	0.7
**HE/TE-EC1**	44.3	44.3	10	0.7	0.7
**HE/TE-EC2**	39.3	39.3	20	0.7	0.7
**HE/TE-EC3**	34.3	34.3	30	0.7	0.7
**HE/TE-EC4**	29.3	29.3	40	0.7	0.7

EC: experimental resin composite; Bis-GMA: bisphenol A glycidyl methacrylate; TEGDMA: triethylene glycol dimethacrylate; DMAEMA: N,N′-dimethylaminoethyl methacrylate; CQ: camphoroquinone.

## Data Availability

The data presented in this study are available on request from the corresponding author. The data are not publicly available due to IPR considerations.
